# Performance of genomic prediction within and across generations in maritime pine

**DOI:** 10.1186/s12864-016-2879-8

**Published:** 2016-08-11

**Authors:** Jérôme Bartholomé, Joost Van Heerwaarden, Fikret Isik, Christophe Boury, Marjorie Vidal, Christophe Plomion, Laurent Bouffier

**Affiliations:** 1 BIOGECO, INRA, Univ. Bordeaux, 33610 Cestas, France; 2 Biometris, Wageningen University and Research Centre, NL-6700 AC Wageningen, The Netherlands; 3 Department of Forestry and Environmental Resources, North Carolina State University, Raleigh, NC USA; 4 FCBA, Biotechnology and Advanced Silviculture Department, Genetics & Biotechnology Team, 33610 Cestas, France

**Keywords:** Genomic selection, Growth, Multiple generations, *Pinus pinaster*, Progeny validation, Relatedness, Stem straightness

## Abstract

**Background:**

Genomic selection (GS) is a promising approach for decreasing breeding cycle length in forest trees. Assessment of progeny performance and of the prediction accuracy of GS models over generations is therefore a key issue.

**Results:**

A reference population of maritime pine (*Pinus pinaster*) with an estimated effective inbreeding population size (status number) of 25 was first selected with simulated data. This reference population (*n* = 818) covered three generations (G0, G1 and G2) and was genotyped with 4436 single-nucleotide polymorphism (SNP) markers. We evaluated the effects on prediction accuracy of both the relatedness between the calibration and validation sets and validation on the basis of progeny performance. Pedigree-based (best linear unbiased prediction, ABLUP) and marker-based (genomic BLUP and Bayesian LASSO) models were used to predict breeding values for three different traits: circumference, height and stem straightness. On average, the ABLUP model outperformed genomic prediction models, with a maximum difference in prediction accuracies of 0.12, depending on the trait and the validation method. A mean difference in prediction accuracy of 0.17 was found between validation methods differing in terms of relatedness. Including the progenitors in the calibration set reduced this difference in prediction accuracy to 0.03. When only genotypes from the G0 and G1 generations were used in the calibration set and genotypes from G2 were used in the validation set (progeny validation), prediction accuracies ranged from 0.70 to 0.85.

**Conclusions:**

This study suggests that the training of prediction models on parental populations can predict the genetic merit of the progeny with high accuracy: an encouraging result for the implementation of GS in the maritime pine breeding program.

**Electronic supplementary material:**

The online version of this article (doi:10.1186/s12864-016-2879-8) contains supplementary material, which is available to authorized users.

## Background

The use of genome-wide DNA markers to predict genomic estimated breeding values (GEBV), first proposed by Meuwissen et al. [1], has radically changed perspectives in molecular breeding. Breeders now have access to large numbers of single-nucleotide polymorphisms (SNPs). They have therefore focused their efforts on genomic selection (GS), which is based on a large set of markers expected to be in linkage disequilibrium (LD) with every QTL controlling the phenotype of interest. In comparison to classical marker-assisted selection, which uses a small set of well-characterized markers tracing a small number of quantitative trait loci (QTLs), each with a medium-to-large effect, GS offers the possibility of a higher genetic gain per unit of time [2–4]. Thus, with the availability of cost-effective genotyping platforms [5], the use of this approach has become widespread in the breeding of animals [4, 6] and plants [7, 8], including forest trees [9, 10]. GS requires the development of a predictive model with a calibration population for which both genotype and phenotype have been characterized. This model is then used to predict GEBV, from marker genotypes alone, in the targeted breeding populations. As in traditional selection based on estimated breeding values (EBV), prediction accuracy is a key issue in evaluations of the efficiency of GS strategies. The prediction accuracy of GS models is evaluated by assessing the correlation between the GEBV obtained with GS models and the EBV obtained by classical genetic evaluation based on progeny testing. Simulation studies, either general [1, 11–15], or species-based (maize [3], oil palm [16], barley [17], Japanese cedar [18]), have attempted to identify key factors affecting the accuracy of GEBV. In a review on dairy cattle, Hayes et al. [4] highlighted four major factors: i) the heritability of the target trait, ii) the genetic architecture of the trait (number and effect of underlying QTLs), iii) the level of LD between markers and QTLs in the reference and target populations, and iv) the size of the reference population, and the degree to which the reference and target populations are related. The statistical methods used to predict GEBV may also affect the accuracy of this prediction [19], but to a lesser extent.

In forest tree breeding, the duration of a single cycle of selection-recombination is driven by the time at which flowering first occurs (e.g. 7–8 years in maritime pine) and the age at which early indirect selection for mature properties can be carried out (e.g. 10–12 years for total height and stem straightness in maritime pine). A full cycle therefore generally lasts more than two decades. In addition, the low–to-medium heritabilities of most complex traits, such as growth, stem form, and branching characteristics, limit the response to selection, and, thus, the expected genetic gain. GS may overcome these limitations, by decreasing breeding cycle duration and improving selection efficiency/intensity for traits with a low heritability, thereby increasing the efficiency of breeding strategies. Preliminary studies on major plantation forest trees (eucalyptus, spruces and pines) have given encouraging results [9, 10], with accuracies of up to 0.8 (Table 1), despite the low level of LD in these outcrossing species, which have large population sizes [20, 21], and low marker coverage (i.e. a few thousand loci). These studies showed that GS with DNA markers provided accuracies similar to those obtained for classical genetic evaluation with progeny testing (Table 1). Rather than capturing historical LD associations between markers and QTLs, this approach derives its prediction accuracy from better estimations of realized genomic relationships [22, 23]. The relatively small effective population sizes of the reference populations and validation within the same population clearly contributed to higher accuracies. Indeed, lower accuracies (around 0.5) were obtained for larger reference populations [24, 25] or when GS models were applied to target populations different from the reference population [26]. It is important to assess the prediction accuracy of GS models across generations, because recombination may modify marker-allele phases in subsequent generations, and because selection may change allele frequencies [10]. These effects may decrease GS accuracy over generations [11, 27]. The validation of GS models across generations, with assessment of the predictive ability of markers, is essential before the implementation of GS strategies in tree breeding. The marker-trait associations established in “parental” populations (the parents or preceding generations) should be validated in progeny populations (i.e., progeny validation) [28, 29]. To our knowledge, no study on forest tree species has yet used empirical data to address this issue. Indeed, in all the studies listed in Table 1, individuals of the same generation were split into calibration and validation sets for the evaluation of GS models.

**Table 1:** List of genomic selection studies based on real data sets conducted on forest tree species. Studies are listed in chronological order of publication. This study is the last one listed

Species	Population				Genotyping		Traits analyzed	Models	Prediction accuracy	Reference
	Size	Family type	Family size	G	Method	Number of markers				
*Eucalyptus* hybrids	738	43 FS	15 to 23	1	DArT array	3129	Growth, wood properties	RR-BLUP	0.54–0.6	[26]
	920	51 FS	10 to 15	1	DArT array	3564			0.38–0.55	
Loblolly pine	790–840	61 FS	-	1	SNP array	4852	Growth	RR-BLUP	0.63–0.75	[51]
Loblolly pine	951	61 FS	15 ± 2.2	1	SNP array	4825	Growth, tree architecture, wood properties, disease resistance	RR-BLUP, Bayes A, Bayes Cπ, B-LASSO, RR-BLUP B	0.17–0.51	[52]
Loblolly pine	149	13 FS	1 to 34	1	SNP array	3406	Growth, wood properties	RR-BLUP	0.30–0.83	[75]
Loblolly pine	165	9 FS	3 to 37	1	SNP array	3461	Growth	ABLUP, GBLUP	0.37–0.74	[71]
White spruce	1694	214 HS	-	1	SNP array	6385	Growth, wood properties	ABLUP, B-RR, B-LASSO	0–0.44	[24]
White spruce	1748	59 FS	25 to 33	1	SNP array	6932	Growth, wood properties	ABLUP, B-RR, Combined	0.33–0.45	[60]
Loblolly pine	956	61 FS	15 ± 2.2	1	SNP array	4825	Growth, tree architecture	ABLUP, RR-BLUP	0.17–0.51	[57]
Maritime pine	661	191 HS	1 to 13	2	SNP array	2500	Growth, stem straightness	GBLUP, B-RR, B-LASSO	0.09–0.73	[25]
Interior spruce	1126	25 HS	<32	1	GBS	8868–62,198	Growth, wood properties	RR-BLUP, GRR	0.34–0.77	[61]
Interior spruce	769	25 HS	-	1	GBS	34,570–50,803	Growth	RR-BLUP, GRR, Bayes Cπ	0.04–0.55	[76]
Maritime pine	817	35 HS	13 to 34	3	SNP array	4332	Growth, stem straightness	ABLUP, GBLUP, B-LASSO	0.24–0.94	This study

*FS* full-sib family, *HS* half-sib family, *G* number of generations included in the study, *GBS* genotyping-by-sequencing method

Maritime pine (*Pinus pinaster*) is a major forest tree species in south-western Europe. A breeding program based on a recurrent selection strategy was initiated in France in the 1960s [30]. A base population of 635 founders (the G0 trees) was selected from the "Landes" ecotype (an ecotype found in South-West France) for growth (height and circumference) and stem straightness. This population was subjected to two cycles of breeding, testing and selection (i.e. the G1 and G2 generations). The potential of GS for use in maritime pine breeding is currently being evaluated alongside the implementation of a forward selection strategy with pedigree reconstruction [31]. A preliminary investigation based on a population of 661 individuals from the first two generations, with low marker coverage (2500 SNPs, i.e. ~1.39 markers/cM), showed the prediction accuracy of GS models to be about 0.50 for growth and stem straightness [25]. In this study, we first selected a reference population on the basis of the following criteria: i) high performance for the main traits of the breeding program, ii) limited effective population size, and iii) combining the three generations of the maritime pine breeding population. Simulations were carried out to optimize the set of individuals to be genotyped for genomic prediction. Finally, using the reference population with real phenotypic and genotypic data, we aimed: i) to compare the predictive power of SNP markers with that of the pedigree-based method, ii) to investigate the effect on prediction accuracy of pedigree depth and relatedness between the calibration and validation sets, and iii) to investigate the impact of the use of third-generation individuals as a validation set (progeny validation) on the prediction accuracy of GS models.

## Methods

### Design of the reference population

**Fig. 1:**
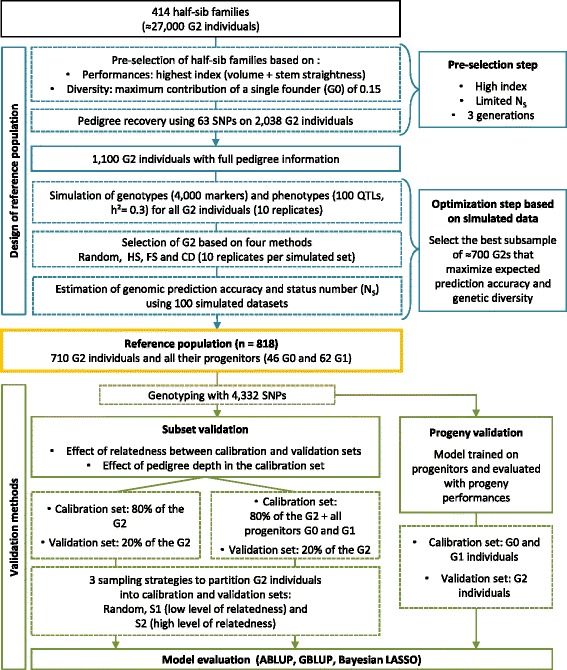
Strategy for selecting the reference population and validation methods for model evaluation. The reference population was designed in two steps. The first was based on breeding value and pedigree information and the second was based on the use of simulated data to optimize the population to be genotyped. The reference population was then used to evaluate the performance of prediction models with different validation methods

The reference population was designed in two steps, as summarized in Fig. 1. A pre-selection step based on pedigree and phenotype information was first applied to G2 individuals and their progenitors. Simulations were then used to select a subset of about 800 individuals (*a priori* on the basis of genotyping constraints) to maximize the expected genomic prediction accuracy.

#### Pre-selection of G2 individuals

G2 individuals were pre-selected in series of polycross trials involving 414 half-sib families (identified mothers were crossed with a pollen mixture) and 27,265 G2 trees. Breeding values based exclusively on maternal pedigree (as the paternal pedigree was unknown) were estimated for height, stem circumference at breast height and stem straightness, in a mixed model framework. Two criteria were used to select a subset of G2 trees: i) an index combining the best linear unbiased predictions (BLUP) for volume and stem straightness (equal weighting) to select the best half-sib family, ii) a maximum of 40 half-sib families with a maximum contribution of a single founder (G0) of 0.15, to prevent the over-representation of a few founders and to give a limited status number (N_S_, an estimate of effective population size). This procedure resulted in the selection of 2038 G2 trees. Pedigree recovery with 63 SNP markers was carried out on these trees to identify the paternal parent and to check the maternal genotype (see Vidal et al. [31] for a description of the methodology). Maternal identity was confirmed and paternal parents (pollen donors) were identified for 1308 G2 individuals. At least one of the grandparents (G0 individuals) was unknown for 208 of the 1308 G2 individuals. We decided to select only G2 trees for which full pedigree information was available. Thus, 1100 G2 trees and their progenitors (78 G1 and 50 G0) were available for the design of the reference population on the basis of simulation data.

#### Simulation to optimize the final selection of the reference population

We used 4000 markers evenly distributed over a 1665 cM composite genetic map of maritime pine [25], including 2965 mapped positions. A gene-dropping algorithm developed in R [32] was used to generate the genotypes of the G1 and G2 offspring. Starting with a set of identified founder haplotypes in generation G0, this algorithm modeled the process of segregation and gamete association over the three generations resulting in known founder alleles at each marker position for each individual in the G2 population. The probability of recombination between adjacent markers was set according to the genetic distance between them. Marker states were assigned randomly to each founder allele, assuming an allele frequency of 0.5 for all markers. The trait of interest was modeled by assigning a non-zero QTL effect, assuming a normal distribution, to 100 random marker positions and setting the environmental error term to give a narrow-sense heritability of 0.3, corresponding to the observed heritability of the target traits [31].

Four methods were applied to the 1100 G2 plants, to establish a reference population of about 800 trees (G0, G1 and G2). In the first method, G2 trees were selected at random (the random method). The second method was based on sampling within the largest maternal half-sib families, with equal numbers of individuals selected from each half-sib family (the HS method). In the third method, G2 trees were sampled from the largest full-sib families, with a maximum of two individuals selected per family (the FS method). For the fourth method, we maximized the mean generalized coefficient of determination (CD method) [33, 34]. The CD method provides a measurement of the expected reliability of predictions based on the pedigree. Briefly, a specified number (eight in our case) of individuals with the highest CD values are removed one-by-one, with the individuals causing the largest decrease in mean CD being retained. This process is repeated until the desired number of individuals remain. We evaluated these four methods by simulating 100 replicates corresponding to 10 different datasets (simulated genotypes and phenotypes), each with 10 different samplings of the G2 generation. Status number (N_S_, [35]) was estimated as ${N}_S=\frac{1}{2}F$, where $F$ is the mean inbreeding value calculated from the realized kinship matrix; see the methods below.

### Phenotypic and genotypic data for the reference population

#### Traits analyzed

The estimated breeding values (EBV) for three different traits — circumference and height at 12 years of age and stem straightness at 8 years of age — were obtained from a meta-analysis based on the TREEPLAN framework [36]. The correlations between circumference and height (Spearman’s correlation coefficient ρ = 0.61, *p* < 0.01) and between circumference and stem straightness (ρ = 0.45, *p* < 0.01) were moderate. A weaker correlation was observed between height and stem straightness (ρ = 0.36 *p* < 0.01, Additional file 1: Figure S1). EBV reliability was generally high (0.97 ± 0.02) for G0 and G1 individuals, and mean EBV reliability for the G2 population was 0.75. Parental effects on the EBV of individuals can be large and may introduce bias into genomic estimated breeding values. The BLUP method shrinks the breeding values towards the mean and reduces the variation. We addressed the issues of bias and reduced heterogeneity by deregressing the EBV of individuals, as suggested by Garrick et al. [37]. We used the heritabilities estimated from TREEPLAN evaluation for deregression: 0.17, 0.32 and 0.26 for circumference, height and stem straightness, respectively. The resulting deregressed breeding values were used as pseudo-phenotypes for the genomic prediction analysis.

#### Genotyping and linkage disequilibrium analysis

The DNA extraction method and the Illumina Infinium array used to genotype the reference population have been described elsewhere [38]. SNP clustering was performed with GenomeStudio (Genotyping module V1.9, Illumina, San Diego, USA), with the manual checking of each SNP. One G2 individual, with a call rate below 0.98 and a 10 % GenCall score below 0.24, was removed. We analyzed 8411 SNP loci: genotyping failed for 2429 (low fluorescence intensity, GenTrain score below 0.35), 1539 were monomorphic and 4443 were polymorphic (52.8 %). The pattern of SNP inheritance was checked with MERLIN [39]. SNPs presenting an aberrant inheritance pattern or for which more than 2 % of values were missing were removed from subsequent analyses. For the remaining 4436 polymorphic SNPs, the mean GenTrain score was 77.7 %, the mean percentage of missing data was 0.05 % and the repeatability, based on eight duplicated genotypes, was greater than 99.9 %. For genomic prediction models, 4332 SNPs were retained on the basis of their minor allele frequency (MAF > 0.01). Genetic location on the *P. pinaster* composite map [40] was determined for 3962 SNPs (91.5 %, Additional file 1: Figure S2), corresponding to a total of 2548 contigs of the *P. pinaster* unigene [41]. The number of markers per linkage group ranged from 279 to 376, with a mean of 330, corresponding to 2.4 SNPs per cM.

The intra-chromosomal LD between markers was calculated as *r*^*2*^ with R software and expressed as a function of the genetic distance between markers. The effect of selection (differentiation between generations), resulting in changes in allele frequencies between generations, was assessed by calculating a fixation index (F_ST_) [42] with the R package *pegas* [43].

### Methods for genomic prediction

Data for genomic prediction models were handled in the R 3.2.2 environment [32] with the R packages *synbreed* [44] and BGLR [45]. The results were visualized with the *ggplot2* package [46].

#### Genetic relationship matrices

Kinship coefficients between individuals of the three-generation pedigree were estimated from pedigree and genomic data. Two expected additive genetic relationship matrices (matrix **A**) based on pedigree were derived. The first, **A**_**P**_, used only data for the maternal parents and corresponds to polymix breeding, in which only the maternal parents are known. For the second (**A**_**F**_), the full pedigree was used. G0 plants were considered to be unrelated and no population structure was identified [20]. In parallel to pedigree-based matrices, a realized genomic relationship matrix (matrix **G**) was also calculated, as described by Van Raden [47].

1 $$ \mathbf{G}=\frac{\left(\mathbf{M}-\mathbf{P}\right)\left(\mathbf{M}-\mathbf{P}\right)\mathit{\hbox{'}}}{2{\displaystyle \sum {p}_i\left(1-{p}_i\right)}} $$

where $\mathbf{M}$ and $\mathbf{P}$ are two matrices of dimension *n* (number of individuals) × *p* (number of markers). $\mathbf{M}$ is the matrix of gene content, with values of −1, 0, and 1, for one homozygote, the heterozygote, and the other homozygote, respectively. $\mathbf{P}$ is the matrix of allele frequencies in the following form $2\left({p}_i-0.5\right)$, where ${p}_i$ is the observed allele frequency at the marker *i* for all individuals. Use of the matrix of minor allele frequency scales $\mathbf{G}$ such that it lies on the scale of the expected additive genetic relationships matrix derived from the pedigree.

#### Statistical models for genomic prediction

We used genomic BLUP and Bayesian LASSO [48] to predict genomic estimated breeding values. The classical genetic evaluation (BLUP) was used to predict genomic estimated breeding values (GEBV) by a mixed model approach, in which the pedigree-based relationship matrix **A** was replaced with the realized genetic relationship matrix **G**. The methods used have been described in detail elsewhere [25], but we summarize them in brief here.

2 $$ \mathbf{y}=\mathbf{1}\mu +\mathbf{Z}\mathbf{u}+\mathbf{e} $$

where $\mathbf{y}$ is a vector of the pseudo-phenotypes (EBV) (dimension $n\kern0.28em \times \kern0.28em 1$), $\mu$ is the overall mean with a vector of **1**, $\mathbf{Z}$ is a design matrix of the random effects with $n\kern0.28em \times \kern0.28em n$ dimensions, $\mathbf{u}$ is the vector of random tree effect ($n\kern0.28em \times \kern0.28em 1$) $\mathbf{u}\sim N\left(0,\;\mathbf{G}{\sigma}_{\mathrm{u}}^2\right)$, and $\mathbf{e}$ is the vector of residuals (dimension $n\times 1$) with expectations $\mathbf{e}\sim N\left(0,\;{\mathbf{I}}_n{\sigma}_{\mathrm{e}}^2\right)$. The diagonal elements of the residual variance covariance matrix **R** are prediction accuracies. For the prediction of GEBV, the **G** matrix derived from DNA markers is used to solve mixed model equations:

3 $$ \left[\begin{array}{c}\hfill \mathbf{1}\mathit{\hbox{'}}\mathbf{1}\hfill \\ {}\hfill \mathbf{Z}\mathit{\hbox{'}}\mathbf{1}\hfill \end{array}\kern1em \begin{array}{c}\hfill \mathbf{1}\mathit{\hbox{'}}\mathbf{Z}\hfill \\ {}\hfill \mathbf{Z}\hbox{'}\mathbf{Z}+{\mathbf{G}}^{-1}\upalpha \hfill \end{array}\right]\left[\begin{array}{c}\hfill \widehat{\boldsymbol{\upmu}}\hfill \\ {}\hfill \widehat{\mathbf{u}}\hfill \end{array}\right]=\left[\begin{array}{c}\hfill \mathbf{1}\mathit{\hbox{'}}\mathbf{y}\hfill \\ {}\hfill \mathbf{Z}\mathit{\hbox{'}}\mathbf{y}\hfill \end{array}\right] $$

where ${\mathbf{G}}^{-1}$ is the inverse of the realized genomic relationship matrix, $\upalpha$ is the residual variance (${\sigma}_e^2$) divided by the variance associated with the random tree effect ${\sigma}_u^2$. This ratio is equal to the sum across loci $2\Sigma {\mathrm{p}}_{\mathrm{i}}\left(1-{\mathrm{p}}_{\mathrm{i}}\right)$ times the ratio ${\sigma}_e^2/{\sigma}_a^2$ where ${\sigma}_a^2$ represents the total genetic variance and ${p}_{\mathrm{i}}$ is the minor allele frequency at the *i*^th^ locus. The ${\mathbf{G}}^{-1}$ matrix was replaced with the ${\mathbf{A}}^{-1}$ matrix for predictions of the breeding values of individuals from expected genetic relationships. GBLUP assumes that markers have the same effects and that each marker has a small effect on the phenotype.

We tested the marker specific shrinkage model, Bayesian LASSO and compared it to GBLUP in terms of GEBV reliability. The linear model has the form: $\mathbf{y}=\boldsymbol{\upmu} +\mathbf{X}\boldsymbol{\upbeta } +\boldsymbol{\upvarepsilon}$, where $\mathbf{X}$ ($n\times p$) is the incidence matrix of markers, $\boldsymbol{\upbeta}$ ($p\times 1$) is the vector of marker effects, and $\boldsymbol{\upvarepsilon}$ ($n\times 1$) is the random residual effect with expectations $\boldsymbol{\upvarepsilon} \sim N\left(0,\;{\mathbf{I}}_n{\sigma}_{\boldsymbol{\upvarepsilon}}^2\right)$. The solutions of marker effects are obtained as

4 $$ {\widehat{\upbeta}}_{\mathrm{L}}=\underset{\upbeta}{ \arg\;\min}\left\{{\left|\mathbf{y}-\mathbf{X}\boldsymbol{\upbeta } \right|}^2+\lambda {\displaystyle {\sum}_{i=1}^p\left|{\upbeta}_i\right|}\right\} $$

The expression outside the curly brackets minimizes the error variance. The shrinkage of markers towards the intercept is marker-specific and regulated by the $\lambda$ parameter [49]. The coefficients of uninformative markers are shrunk to exactly zero, reducing the complexity of the model and this can be used as the basis of a model selection method. A scaled inverse ${\upchi}^{-2}$ prior with ${df}_{\varepsilon }$ degrees of freedom and scale parameter ${S}_{\varepsilon }$ was assigned as a flat prior to residual effect as ${\sigma}_{\boldsymbol{\upvarepsilon}}^2\sim {\upchi}^{-2}\left({\sigma}_{\boldsymbol{\upvarepsilon}}^2,\;{S}_{\varepsilon}\right)$. We used the same priors and rate parameters as Isik et al. [25] for the Bayesian LASSO regression coefficients. The vector ${\boldsymbol{\upbeta}}_{\mathrm{L}}$ is assumed to have a multivariate normal distribution with marker-specific prior variances with expectations ${\boldsymbol{\upbeta}}_{\mathrm{L}}\sim \mathrm{N}\left(0,\mathbf{T}\Big({\sigma}_{\mathrm{e}}^2\right)\Big)$, where $\mathbf{T}=\mathrm{diag}\left({\tau}_1^2,\dots, {\tau}_q^2\right)$. We assigned ${\tau}_j^2$ parameters independently and used identically distributed exponential priors, ${\tau}_j^2\sim Exp\left({\lambda}^2\right)$ for $j=1,\dots, q$, where parameter ${\uplambda}^2$ is given a gamma prior distribution with hyper-parameters $r$ (shape) and $\updelta$ (rate), giving ${\uplambda}^2\sim \mathrm{gamma}\left(r,\updelta \right)$ [48, 50].

#### Definition of the calibration and validation sets and model evaluation

Based on the reference population; two different validation methods were used to evaluate the effect of the structure of the calibration set on genomic prediction accuracies: subset validation and progeny validation (Fig. 1).

The subset validation method, in which the G2 population was split into calibration and validation sets, evaluated the effect of the relatedness of the calibration and validation sets on prediction accuracy. Three different sampling strategies were used to sample 20 % of the G2 population to form the validation set: i) random selection of G2 trees (random), ii) selection of G2 trees from the same half-sib families, to obtain a low level of relatedness between the calibration and validation sets (S1), iii) sampling of G2 trees from different full-sib families, to obtain a high level of relatedness between the calibration and validation sets (S2). For each sampling strategy, two types of calibration sets were used to evaluate the effect of pedigree depth. The first was the remaining 80 % of the G2 population and the second was the remaining 80 % of the G2 population plus all progenitors (G0 and G1). Model fit statistics were obtained for 100 replications for each scenario.

In addition to subset validation (different sampling approaches applied to G2 trees), we performed progeny validation to evaluate the prediction accuracy of GS models over generations. The individuals of the G0 and G1 generations were used as the calibration set and the individuals of the G2 generation were used as the validation set. This second validation method was used to assess the accuracy of genomic prediction models across generations, with the model trained on ancestral generations (Gn, Gn-1, etc.) and validated on progeny generation (Gn + 1). The prediction accuracy of GS models was estimated as the coefficient of correlation between the genomic estimate breeding values (GEBV) of the validation set and the EBV obtained by TREEPLAN evaluation. The prediction bias was calculated as the slope of the regression line between EBV and GEBV. A slope of b > 1 indicates deflation and a slope of b < 1 indicates inflated predictions.

## Results

### Design of the reference population

**Fig. 2:**
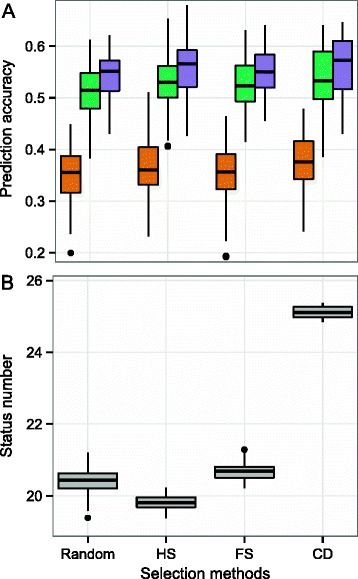
Prediction accuracy (**a**) and status number (**b**) based on simulated data. Results are given for four methods for selecting G2 individuals (Random, HS: half-sib family, FS: full-sib family and CD: coefficient of determination). The prediction accuracy was calculated as Pearson’s correlation coefficient for the relationship between GEBV and true breeding values for the validation set assessed by the cross-validation method. The results obtained with A_P_BLUP are in orange, those obtained with A_F_BLUP are in green, and those obtained with GBLUP are shown in purple. A Tukey boxplot is used to represent the data

For all pre-selection methods, (Random, HS, FS and CD), use of the full-pedigree information (matrix **A**_**F**_) substantially increased the prediction accuracy of GS models (*p* < 0.05) over that for the partial pedigree based only on maternal information (matrix **A**_**P**_, Fig. 2a). Small but significant increases in prediction accuracy (0.03 on average, *p* < 0.05) were achieved by using GBLUP rather than A_F_BLUP. For example, for the HS selection method, the mean accuracies of genomic predictions were 0.53 for A_F_BLUP and 0.56 for GBLUP (Additional file 1: Table S1). For all relationship matrices, the CD method performed significantly better than the other three methods (Fig. 2a). However, the differences were small: the mean prediction accuracies for GBLUP were 0.54, 0.56, 0.55 and 0.56 for the Random, HS, FS and CD selection methods, respectively. Status number depended on the selection method used (Fig. 2b). The highest N_S_ value was obtained for the CD selection method (25.1 on average). This value was significantly higher than those for the HS (19.8), FS (20.7) or Random (20.4) methods (Additional file 1: Table S1). We therefore used the CD method to select the reference population, as it gave the highest prediction accuracy and N_S_.

### Characterization of the reference population

**Fig. 3:**
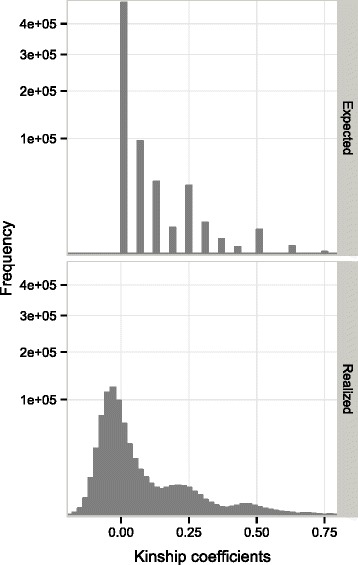
Comparison between expected and realized genetic relationship coefficients. Expected additive genetic relationships from the pedigree (*top panel*) and realized genetic relationships from SNP markers (*bottom panel*), for the reference population

The reference population selected by the CD method comprised 818 individuals from the three generations (Additional file 1: Figure S3): 710 G2 trees and all their progenitors (62 G1 and 46 G0). The G2 individuals came from 35 maternal half-sib families, corresponding to 355 full-sib families. The number of individuals per half-sib family ranged from 13 to 34, with a mean value of 22.2. As expected, given the low level of relatedness in the population (founder G0 trees are not related), a large majority of the kinship coefficients estimated from the pedigree were zero. The coefficients obtained were grouped into 11 classes and ranged from 0 to 0.75 (Fig. 3). By using markers, we were able to estimate the proportion of the genome shared by different individuals. The relationships predicted from markers were more consistent with the actual relationships than the expected genetic relationships derived from the pedigree (Fig. 3). Unlike the expected genetic relationships derived from the pedigree, the realized genetic relationships in the **G** matrix were continuously distributed, with values between −0.18 and 0.77 (Fig. 3). Some of the realized genetic relationships were negative, suggesting that some individuals shared fewer markers than expected on the basis of allele frequencies. Similarly, some pairs of coefficients were positive and close to zero due to the sharing of a larger number of alleles than expected from allele frequencies.

The extent of LD in the reference population was estimated by calculating *r*^*2*^ from 3962 markers mapped onto the *P. pinaster* composite map. A rapid decrease in intra-chromosomal LD was observed for an inter-marker distance of about 5 cM on all linkage groups (Additional file 1: Figure S4). The overall LD was close to zero (average *r*^*2*^ = 0.016) and only a few marker pairs (0.5 %) had *r*^*2*^ values greater than 0.4. Most of the markers concerned (96.5 %) were physically linked (on the same contig) or genetically linked (less than 5 cM apart on the composite map). The remaining markers displaying high levels of LD (2.5 %) probably reflected a bias in composite linkage map construction rather than true long-distance LD, as suggested by their positions on component maps. Changes in allele frequencies were observed between G0 and G1, with an F_ST_ value greater than 0.05 for 19 SNPs, mostly located on chromosomes 5, 6, 9 and 12 (Additional file 1: Figure S5). By contrast, no difference was observed between G1 and G2. Overall, almost no differentiation was found between generations, with a global F_ST_ <0.01 between G0 and G1 and between G1 and G2.

### Prediction accuracy of genomic selection models for the reference population

#### Effect of calibration set structure on accuracy

The mean prediction accuracies of models using 80 % of the G2 for the calibration set ranged from 0.52 to 0.87, depending on the trait and the scenario considered (Table 2). When G0 and G1 trees were added to the calibration set, mean prediction accuracies ranged from 0.66 to 0.91. Whatever the calibration set or trait considered, mean prediction accuracies for models using only pedigree information (ABLUP) were higher than those for models using marker information (GBLUP or B-LASSO, Additional file 1: Figure S6). This difference was larger (up to 0.1 larger on average) for the scenario including the progenitors of the G2 trees (generations G0 and G1) in the calibration set, suggesting that it is important to use a deep pedigree to increase prediction accuracy. As expected, when the level of relatedness between the calibration and validation sets was low (S1), mean prediction accuracy was lower than that for random sampling or S2 sampling (Table 2, Additional file 1: Figure S6). Overall, the prediction accuracy of S2 was about 0.17 lower than that for S1, for all traits and all models, if only G2 trees were used for the calibration set. Inclusion of the progenitors of the G2 trees in the calibration set resulted in a much smaller difference in prediction accuracy between S1 and S2 (maximum difference of 0.03). For random or S2 sampling, the gain in prediction accuracy achieved by adding the progenitors of G2 trees to the calibration set was smaller (0.03 and 0.02 on average, for random and S2 sampling, respectively) than that for S1 sampling (0.12 on average). However, not all traits followed this general trend. For example, the increase in prediction accuracy for stem straightness was close to zero when progenitors of the G2 trees were added to the calibration population, for the GBLUP and B-LASSO models (Table 2, Additional file 1: Figure S6).

**Table 2:** Comparison of prediction accuracies across three sampling and two calibration strategies. Three sampling strategies for the selection of 20 % of the G2 population as the validation set were applied: random, S1: between half-sib families and S2: within full-sib families. Two calibration strategies were used for each sampling strategy. For predictions for the 20 % of the G2 population selected, we used the remaining 80 % of the G2 plus their progenitors (G0 and G1) as the calibration set. The mean prediction accuracy (and range) for models based on pedigree information (ABLUP) and marker information (GBLUP and B-LASSO), and the results for the three traits studied (tree diameter, height and stem straightness) are presented

		Calibration set: 80 % of the G2			Calibration set: 80 % of the G2 + G0/G1		
		ABLUP	GBLUP	B-LASSO	ABLUP	GBLUP	B-LASSO
Circumference	Random	0.78 (0.68–0.85)	0.73 (0.62–0.80)	0.72 (0.62–0.80)	0.83 (0.79–0.89)	0.74 (0.67–0.81)	0.74 (0.67–0.81)
	S1	0.55 (0.34–0.74)	0.52 (0.24–0.67)	0.52 (0.24–0.67)	0.81 (0.65–0.89)	0.69 (0.51–0.81)	0.69 (0.51–0.81)
	S2	0.80 (0.73–0.85)	0.74 (0.67–0.81)	0.74 (0.67–0.80)	0.84 (0.8–0.89)	0.75 (0.68–0.84)	0.75 (0.68–0.82)
Height	Random	0.68 (0.54–0.78)	0.66 (0.56–0.77)	0.66 (0.56–0.77)	0.75 (0.66–0.82)	0.68 (0.6–0.76)	0.68 (0.59–0.75)
	S1	0.58 (0.46–0.77)	0.58 (0.43–0.75)	0.58 (0.38–0.74)	0.74 (0.63–0.87)	0.67 (0.54–0.79)	0.66 (0.53–0.79)
	S2	0.70 (0.6–0.77)	0.69 (0.60–0.76)	0.68 (0.59–0.76)	0.75 (0.66–0.83)	0.70 (0.59–0.79)	0.69 (0.59–0.79)
Stem straightness	Random	0.86 (0.8–0.90)	0.81 (0.75–0.86)	0.82 (0.76–0.86)	0.90 (0.86–0.94)	0.82 (0.74–0.88)	0.82 (0.75–0.88)
	S1	0.67 (0.51–0.79)	0.65 (0.48–0.77)	0.66 (0.48–0.77)	0.88 (0.78–0.93)	0.77 (0.62–0.87)	0.77 (0.63–0.87)
	S2	0.87 (0.84–0.91)	0.81 (0.77–0.87)	0.81 (0.77–0.88)	0.91 (0.88–0.94)	0.80 (0.76–0.85)	0.80 (0.76–0.86)

#### Predictive value of markers across generations with progeny validation

**Fig. 4:**
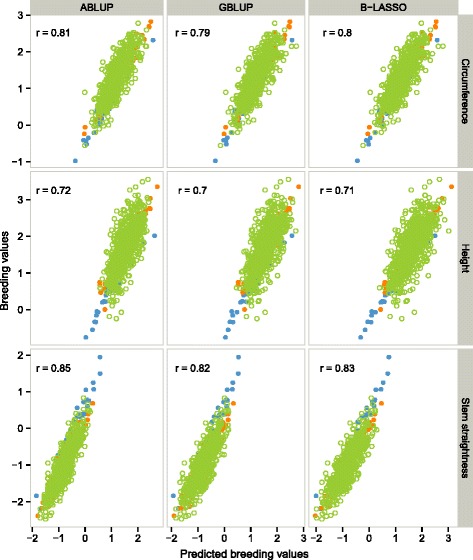
Relationship between predicted breeding values (*x*-axis) and empirical breeding values (*y*-axis) for the progeny validation method. The three traits (circumference, height and stem straightness) and three different models (ABLUP, GBLUP and B-LASSO) are represented. The prediction accuracy (*r*) of genomic prediction models evaluated on the validation set (G2 genotypes are shown as open green circles) is indicated. Closed circles represent the calibration set with G0 genotypes (*n* = 46) in blue and G1 genotypes (*n* = 62) in orange

The prediction accuracies of models using only the G0 and G1 genotypes for the calibration set and only G2 for the validation set ranged from 0.70 to 0.85, depending on the trait and the method considered (Fig. 4, Additional file 1: Table S2). For all traits, ABLUP had a similar or slightly higher (up to 0.03) prediction accuracy than genomic predictions (GBLUP and B-LASSO). For all models and all three traits (except for circumference with B-LASSO model), a bias greater than one was observed, indicating that GEBV was overestimated relative to EBV. The B-LASSO model had the lowest bias: 0.99, 1.07 and 1.06 for circumference, height and stem straightness, respectively. Conversely, ABLUP had the highest bias, at 1.15, 1.22 and 1.36 for circumference, height and stem straightness, respectively (Fig. 4, Additional file 1: Table S2).

## Discussion

### Factors affecting the prediction accuracy of GS models

Our reference population was specifically designed to maximize prediction accuracy given the available genetic material. By contrast to previous GS studies on forest trees, we used simulation to select individuals on the basis of an explicit criterion maximizing the expected prediction accuracy for the population. As a result, we obtained medium-to-high prediction accuracies for all three traits studied (0.52 to 0.91), consistent with published results for forest tree species (Table 1). Indeed, despite differences in species, population structure and GS models between studies, similar accuracies were reported for height in eucalyptus hybrids [26] and loblolly pine [51], with values ranging from 0.66 to 0.79 for eucalyptus and from 0.64 to 0.74 for loblolly pine, depending on effective population size (N_e_) or environment. No clear trend was observed for the relationship between accuracy and trait heritability. Using a large number of traits, resulting in a wider range of heritability, Resende et al. [52] reported a strong correlation (*R*^*2*^ = 0.79) between predictive ability and narrow-sense heritability. Compared to a previous study on maritime pine with a broader genetic basis [25], our results showed higher prediction accuracies on the same traits. The smaller effective population size in this study, measured as status number (N_S_ = 25), than in a previous study (N_S_ ≈ 100) and the inclusion of multiple generations might account for the higher prediction accuracies in this study. Indeed, effective population size, which is directly related to level of LD and relatedness in populations, is known to be an important factor determining GS accuracy [10]. The importance of effective population size for prediction accuracy was highlighted by Resende, MDV et al. [26], in a study using empirical datasets for *Eucalyptus* with contrasting effective population sizes: N_e_ = 11 and N_e_ = 51.

The level of relatedness between the calibration and validation sets also affected GEBV estimates. We found a 0.17 difference in prediction accuracy between low (S1) and high (S2) levels of relatedness. Our findings are consistent with previous results for white spruce [24] and mice [53]. Similar results have been reported for eucalyptus, in which the GS model was applied to populations other than that used to build the prediction model [26]. In cases of a higher marker density, yielding a more stable linkage phase between markers and QTLs across populations, population-specific models have also been described [54]. Similarly, Hayes et al. [55] reported an accuracy close to zero for the use of models developed for the Holstein breed to predict GEBVs for the Jersey breed, and *vice versa*. Thus, in the presence of short-distance LD, as in the maritime pine population in this study, the relatedness of the calibration and validation sets may be the main driver of prediction accuracy [22].

### Comparison between pedigree- and marker-based models

Given all the possible mechanisms separating genomic variants, such as SNPs, from phenotype expression and the efforts required to identify them, one of the main issues in GS studies is demonstrating the predictive value of markers relative to conventional BLUP. In this study, regardless of the scenario used, the model using pedigree information (ABLUP) had a higher prediction accuracy than marker-based models. The marker density (2.4 SNPs per cM) used to predict GEBV may account for this difference. Indeed, simulation studies have suggested that there may be a positive asymptotic relationship between marker density and prediction accuracy [14, 22, 56]. Using a deterministic approach, Grattapaglia [10] showed that the minimal density at which marker-based models achieve accuracies similar to those of ABLUP was 2–3 SNPs per cM for an effective population size below 60. In addition, our reference population selection strategy may also have reduced the additional gain of information provided by molecular markers relative to the pedigree. Indeed, one of the steps in the selection process was pedigree recovery, which improved the estimation of BVs [31]. Indeed, Munoz et al. [57] reported that using the G matrix to correct the pedigree and re-estimate EBVs increased prediction accuracy. In the presence of pedigree errors, which are frequently reported in tree breeding programs [31, 58, 59], the differences in prediction accuracy between ABLUP and GBLUP observed in previous GS studies may be biased. However, our results are consistent with previous findings for forest trees based on simulated [14, 18] or empirical [24, 26, 60, 61] data, with conventional BLUP having an accuracy similar to or slightly higher than that of GS models, particularly for traits with a low heritability. The genetic gain per unit of time of the GS approach over conventional BLUP would therefore be dependent solely on the decrease in breeding cycle length. This decrease in breeding cycle length raises questions about the loss of genetic variation and the maintenance of long-term genetic gain relative to conventional BLUP [62–64].

### GS accuracy over generations

This study is novel because, unlike previous empirical studies on forest trees, we assessed the predictive value of markers across generations, rather than splitting a single population in two for model development and validation [27]. GS in forest trees is likely to be used to select progeny within families without the need for progeny testing, to reduce breeding cycle length. In this case, GS evaluation must be carried out with the progeny population. During the breeding process, recombination between haplotypes should decrease the marker-QTL linkage phase. As a result, prediction accuracy would be expected to decrease over generations [11, 17]. In this study, we assessed the predictive value of the markers, using the parents (G1) and grandparents (G0) as the training set, with validation of the model on the descendants (G2). Interestingly, prediction accuracy remained high (0.70 to 0.85, depending on the trait considered) in the validation set. These accuracies were very similar to those estimated by subset validation with a high level of relatedness between the calibration and validation sets (S2), although the calibration set was larger in this second case (567 vs. 108). These results are consistent with those of Sallam et al. [28] for a five-generation population of barley and with findings for oat breeding lines and cultivars from distant generations [65]. Indeed, both studies reported consistent prediction accuracies over generations for most traits. In sugar beet, Hofheinz et al. [29] reported that prediction accuracy was similar across generations for sugar content but that it decreased by 0.4 for molasses loss. These results suggest that the predictive value of markers across generations is sensitive to the genetic architecture of the trait. Marker density was low in this study and in the three studies described above. However, a larger number of markers should become available in the near future, because further decreases in the cost of genotyping are anticipated. Additional markers will, therefore, probably be included in GS models over generations to maintain the accuracy of GEBV at an operational level [64].

When progenitors (G0 and G1) of the G2 population were included in calibration models, differences in prediction accuracy between low (S1) and high (S2) levels of relatedness were less than 0.03. Moreover, a slight increase in prediction accuracy was observed for all scenarios, highlighting the importance of genotyping the ancestral populations, which are generally conserved in tree breeding programs, to increase prediction accuracy. Simulation studies have also highlighted the importance of including multiple generations in the calibration set, to update the prediction equation [18, 66]. Indeed, a simulation study carried out on *Cryptomeria japonica* trees generated over a period of 60 years showed that GS outperformed phenotypic selection only if the GS model was updated [18]. Sallam et al. [28] reported contrasting results for empirical data from barley, for which the inclusion of previous generations increased prediction accuracy for some traits, but decreased it for others.

### Prospects for the use of GS in the maritime pine breeding program

The maritime pine breeding program follows a recurrent selection scheme, with breeding value estimated from polycross and bi-parental progeny trials. The genetic gain achieved in the released varieties over the first two generations was estimated at 30 % for both growth and stem straightness. The improved varieties generated by this program in the future will need to be adapted to predict changes in climate, pest and disease outbreaks and the demand for diversified wood-based products. The major challenge faced in this breeding program will therefore be the integration of new traits to deliver suitable varieties. With the rapid decrease in genotyping costs and the promising results obtained for forest trees (Table 1), GS could prove an essential tool for addressing these challenges and overcoming the limitations of marker-assisted selection [27, 67]. One of the main advantages of GS is that it can be included in the framework of current genetic evaluation. Indeed, the currently used pedigree-based BLUP method could be replaced with the "single-step" GS strategy [68] with only minimal changes. This strategy is based on the integration of both genotyped and ungenotyped individuals into the genetic evaluation through a hybrid pedigree-genomic relationship matrix [69, 70]. As an increasing number of individuals are being genotyped for higher densities of markers, the information obtained could be used, to decrease the error rate in pedigrees. By eliminating pedigree errors and adding more information (concerning the father), this method should increase the accuracy of genetic evaluation [31, 57]. In addition, GS on the progeny population should make it possible to capture the Mendelian segregation effect in families. In forest trees, crossing can generate large numbers of offspring. In the absence of GS models, all the offspring are considered to have the same mid-parent BV at the seed or seedling stage (before progeny testing) [71]. The challenge is thus to select the superior plants without progeny testing. GS models can meet this challenge, by selecting a subset of progeny on the basis of their GEBV. This should greatly shorten the breeding cycle and decrease the costs of progeny testing, which is expensive and time-consuming for forest trees. Furthermore, a more complete knowledge of the genotype of all candidates for selection should improve the management of genetic diversity and inbreeding depression. However, shortening of the breeding cycle in maritime pine should combine GS with artificial flower induction by top-grafting, as in loblolly pine [51], or by growth regulators, as suggested for *Eucalyptus* [72] and white spruce [24]. These techniques have already been successfully implemented in these species [73, 74], but not yet in maritime pine.

## Conclusion

We selected a reference population covering three generations, with a limited status number (N_S_ = 25) and a marker density of 2.5 SNPs per cM, for assessment of the prediction accuracy of GS models within and across generations. We studied three major traits used in maritime pine breeding: circumference, height and stem straightness. These three traits have low heritabilities, from 0.17 to 0.32. Prediction accuracies of up to 0.85 were obtained with progeny validation, confirming the potential of GS to predict progeny performance for low-heritability traits. However, the pedigree-based model had prediction accuracies similar to or greater than that of marker-based models. The optimization of current breeding strategies based on polymix breeding will therefore be required to enhance the potential of the GS approach in the maritime pine breeding program.

## Abbreviations

A, expected additive genetic relationship matrix; B-LASSO, Bayesian least absolute shrinkage and selection operator; BLUP, best linear unbiased prediction; CD, coefficient of determination; EBV, estimated breeding value; FS, full-sibs; F_ST_, Fixation index; G, realized genomic relationship matrix; G0, G1 and G2, generations of the breeding program; GEBV, genomic estimated breeding value; GS, genomic selection; HS, half-sibs; LD, linkage disequilibrium; N_e_, effective population size; N_S_, status number; QTL, quantitative trait loci

## Additional file

Additional file 1: Figure S1. Scatter plots (lower diagonal), histograms (diagonal) and correlations, with their significance (H_0_: $r=0$, upper diagonal), between breeding values for the traits: circumference, height and stem straightness. Individuals from the G0 generation are in blue, G1 in orange and G2 in green. **Figure S2.** *P. pinaster* composite map [40]. Markers in red correspond to 3965 of the 4335 SNPs used for genomic prediction analysis. **Figure S3.** Pedigree of the 818 trees comprising the reference population (N_S_ = 25) with the following frequency for each generation: G0 = 46, G1 = 62 and G2 = 710. Links in purple represent mother–progeny relationships and those in orange represent father–progeny relationships. Pedigree Viewer software was used to represent the relatedness between individuals from the three generations. **Figure S4.** Pairwise linkage disequilibrium (LD) based on 3962 single-nucleotide polymorphisms mapped onto the twelve linkage groups (LG) of *P. pinaster*. Only loci with minor allele frequencies greater than 0.01 were included in the analysis. **Figure S5.** Distribution of the fixation index (F_ST_) over the 12 chromosome of maritime pine. The top panel represents the F_ST_ between G0 and G1 and the bottom panel represents the F_ST_ between G1 and G2. **Figure S6.** Comparison of prediction accuracies across three sampling and two calibration strategies. Three sampling strategies for the selection of 20 % of the G2 population for use as the validation set were used: random, S1: between half-sib families and S2: within full-sib families. Two calibration scenarios were used for each sampling strategy. For predictions for the 20 % of the G2 population selected, we used the remaining 80 % of the G2 (in green) plus their progenitors (G0 and G1, in blue) as the calibration set. The results for models based on pedigree information (ABLUP) and marker information (GBLUP and B-LASSO), and the results for the three traits studied (tree diameter, height and stem straightness) are presented. The data are represented in a Tukey boxplot. **Table S1.** Prediction accuracy and status number (N_S_) for four methods of selecting G2 individuals. Prediction accuracy was estimated with three relationship matrices (A_P_, A_F_ and G). Mean values (standard deviation in parentheses) are based on 100 replicates per model. The four selection methods were: Random, HS: half-sib family, FS: full-sib family and CD: coefficient of determination. **Table S2.** Prediction accuracy and bias for the use of the progeny population for validation (calibration set = G0 and G1, validation set = G2). The results for the ABLUP, GBLUP and Bayesian LASSO (B-LASSO) models and for the traits tree circumference, height and stem straightness are presented. (PDF 1725 kb)
