# Supplementary material for: Performance of genomic prediction within and across generations in maritime pine
Source: BMC Genomics. 2016 Aug 11;17:604. doi: 10.1186/s12864-016-2879-8 (PMC4981999; doi:10.1186/s12864-016-2879-8)
Supplement: Additional file 1: Figure S1. — Scatter plots (lower diagonal), histograms (diagonal) and correlations, with their significance (H0: $r=0$r=0, upper diagonal), between breeding values for the traits: circumference, height and stem straightness. Individuals from the G0 generation are in blue, G1 in orange and G2 in green. Figure S2. P. pinaster composite map [40]. Markers in red correspond to 3965 of the 4335 SNPs used for genomic prediction analysis. Figure S3. Pedigree of the 818 trees comprising the reference population (NS = 25) with the following frequency for each generation: G0 = 46, G1 = 62 and G2 = 710. Links in purple represent mother–progeny relationships and those in orange represent father–progeny relationships. Pedigree Viewer software was used to represent the relatedness between individuals from the three generations. Figure S4. Pairwise linkage disequilibrium (LD) based on 3962 single-nucleotide polymorphisms mapped onto the twelve linkage groups (LG) of P. pinaster. Only loci with minor allele frequencies greater than 0.01 were included in the analysis. Figure S5. Distribution of the fixation index (FST) over the 12 chromosome of maritime pine. The top panel represents the FST between G0 and G1 and the bottom panel represents the FST between G1 and G2. Figure S6. Comparison of prediction accuracies across three sampling and two calibration strategies. Three sampling strategies for the selection of 20 % of the G2 population for use as the validation set were used: random, S1: between half-sib families and S2: within full-sib families. Two calibration scenarios were used for each sampling strategy. For predictions for the 20 % of the G2 population selected, we used the remaining 80 % of the G2 (in green) plus their progenitors (G0 and G1, in [file 12864_2016_2879_MOESM1_ESM.pdf]

Supplemental Figures and Tables

Performance of genomic prediction within and across generations in maritime pine

Jérôme BARTHOLOMÉ, Joost VAN HEERWAARDEN, Fikret ISIK, Christophe BOURY, Marjorie VIDAL, Christophe PLOMION, Laurent BOUFFIER*

*For correspondence
Address: INRA, UMR1202, BIOGECO, Cestas F-33610, France
Email: bouffier@pierroton.inra.fr

Article acceptance date:

The following Supporting Information is available for this article:

Figure S1 Scatter plots (lower diagonal), histograms (diagonal) and correlations, with their significance (H0: $r = 0$, upper diagonal), between breeding values for the traits: circumference, height and stem straightness. Individuals from the G0 generation are in blue, G1 in orange and G2 in green.

Figure S2 *P. pinaster* composite map [40]. Markers in red correspond to 3965 of the 4335 SNPs used for genomic prediction analysis.

Figure S3 Pedigree of the 818 trees comprising the reference population (NS=25) with the following frequency for each generation: G0=46, G1=62 and G2=710. Links in purple represent mother–progeny relationships and those in orange represent father–progeny relationships. Pedigree Viewer software was used to represent the relatedness between individuals from the three generations.

Figure S4 Pairwise linkage disequilibrium (LD) based on 3,962 single-nucleotide polymorphisms mapped onto the twelve linkage groups (LG) of *P. pinaster*. Only loci with minor allele frequencies greater than 0.01 were included in the analysis.

Figure S5 Distribution of the fixation index (FST) over the 12 chromosome of maritime pine. The top panel represents the FST between G0 and G1 and the bottom panel represents the FST between G1 and G2.

Figure S6 Comparison of prediction accuracies across three sampling and two calibration strategies. Three sampling strategies for the selection of 20% of the G2 population for use as the validation set were used: random, S1: between half-sib families and S2: within full-sib families. Two calibration scenarios were used for each sampling strategy. For predictions for the 20% of the G2 population selected, we used the remaining 80% of the G2 (in green) plus their progenitors (G0 and G1, in blue) as the calibration set. The results for models based on pedigree information (ABLUP) and marker information (GBLUP and B-LASSO), and the results for the three traits studied (tree diameter, height and stem straightness) are presented. The data are represented in a Tukey boxplot.

Table S1 Prediction accuracy and status number (NS) for four methods of selecting G2 individuals. Prediction accuracy was estimated with three relationship matrices (AP, AF and G). Mean values (standard deviation in parentheses) are based on 100 replicates per model. The four selection methods were: Random, HS: half-sib family, FS: full-sib family and CD: coefficient of determination.

Table S2 Prediction accuracy and bias for the use of the progeny population **for** validation (calibration set = G0 and G1, validation set= G2). The results for the ABLUP, GBLUP and Bayesian LASSO (B-LASSO) models and for the traits tree circumference, height and stem straightness are presented.

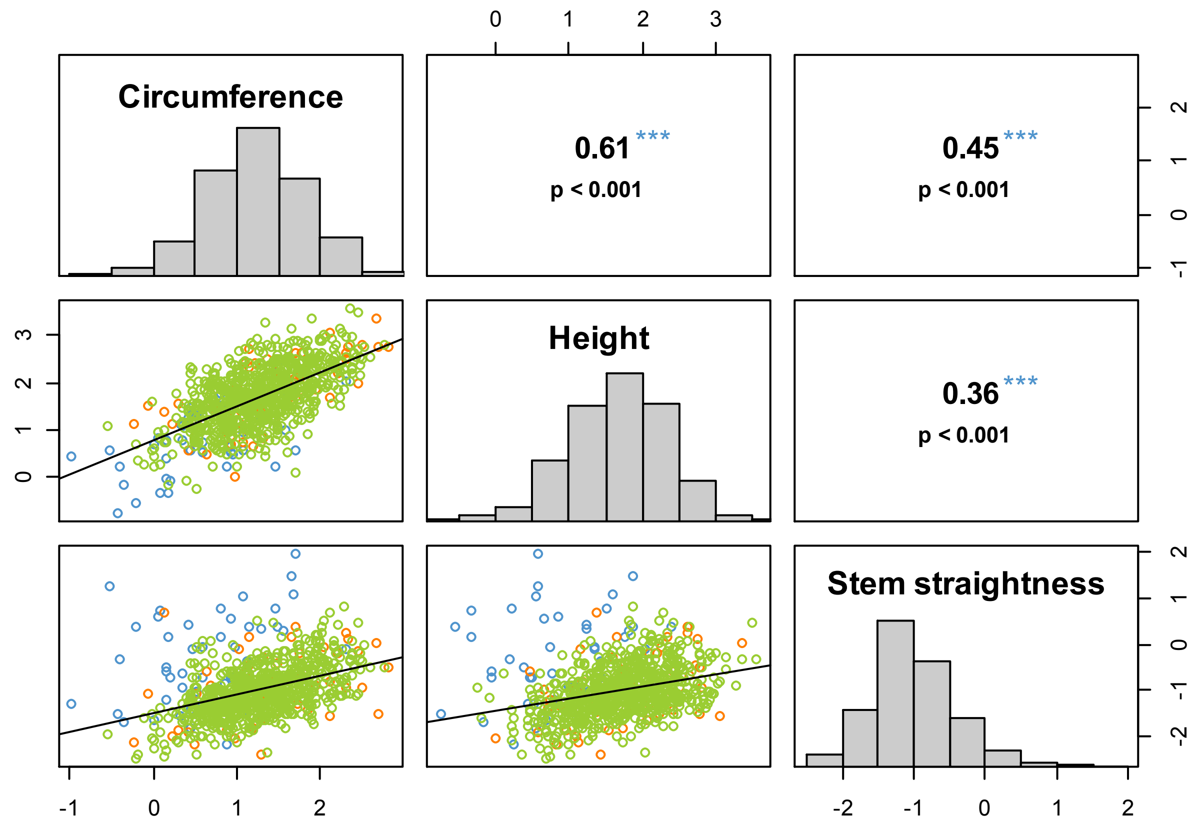

Figure S1 Scatter plots (lower diagonal), histograms (diagonal) and correlations, with their significance (H_0: $r = 0$, upper diagonal), between breeding values for the traits: circumference, height and stem straightness. Individuals from the G0 generation are in blue, G1 in orange and G2 in green.

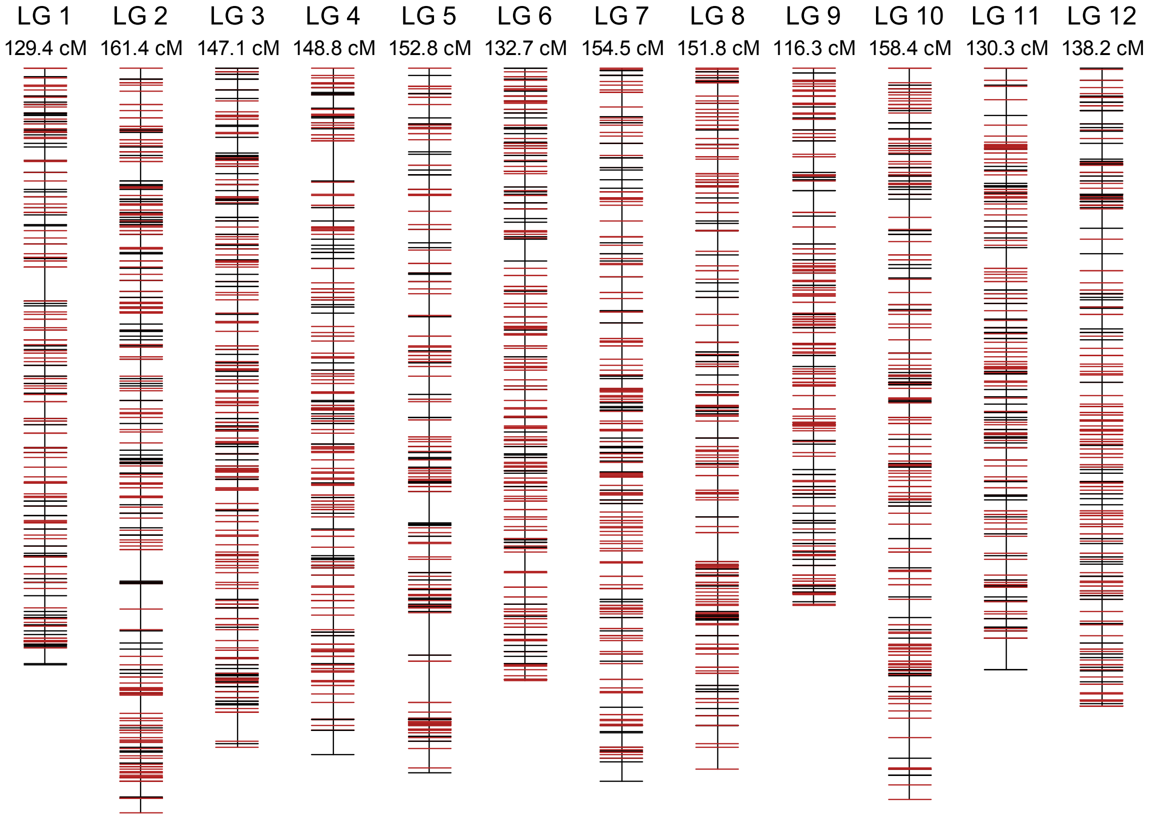

Figure S2 *P. pinaster* composite map [40]. Markers in red correspond to 3965 of the 4335 SNPs used for genomic prediction analysis.

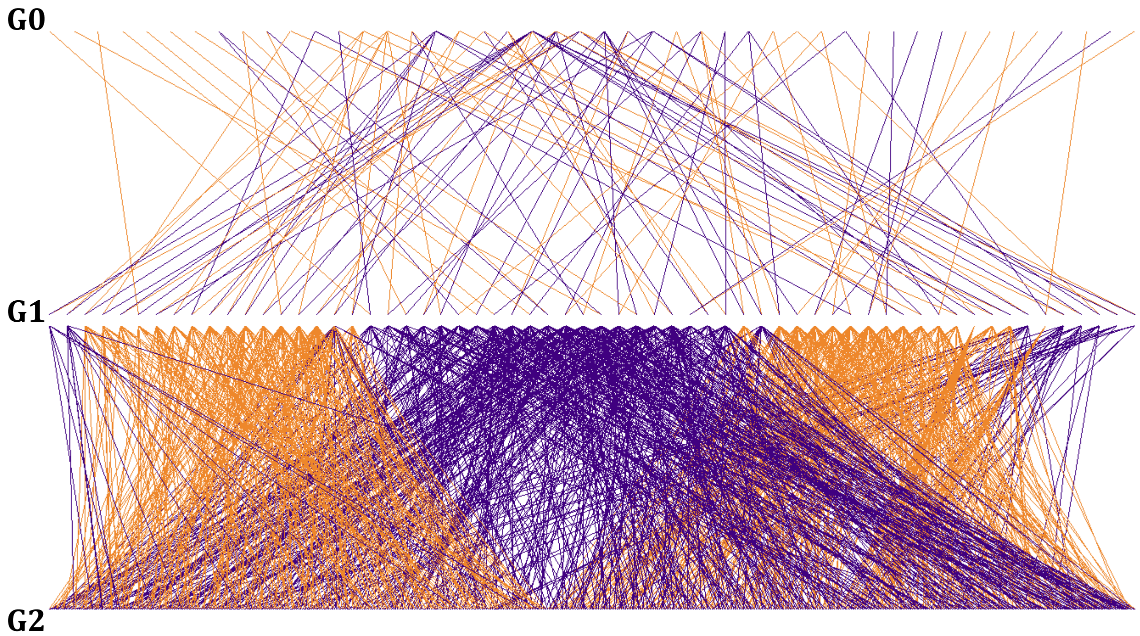

Figure S3 Pedigree of the 818 trees comprising the reference population (N_S=25) with the following frequency for each generation: G0=46, G1=62 and G2=710. Links in purple represent mother–progeny relationships and those in orange represent father–progeny relationships. Pedigree Viewer software was used to represent the relatedness between individuals from the three generations.

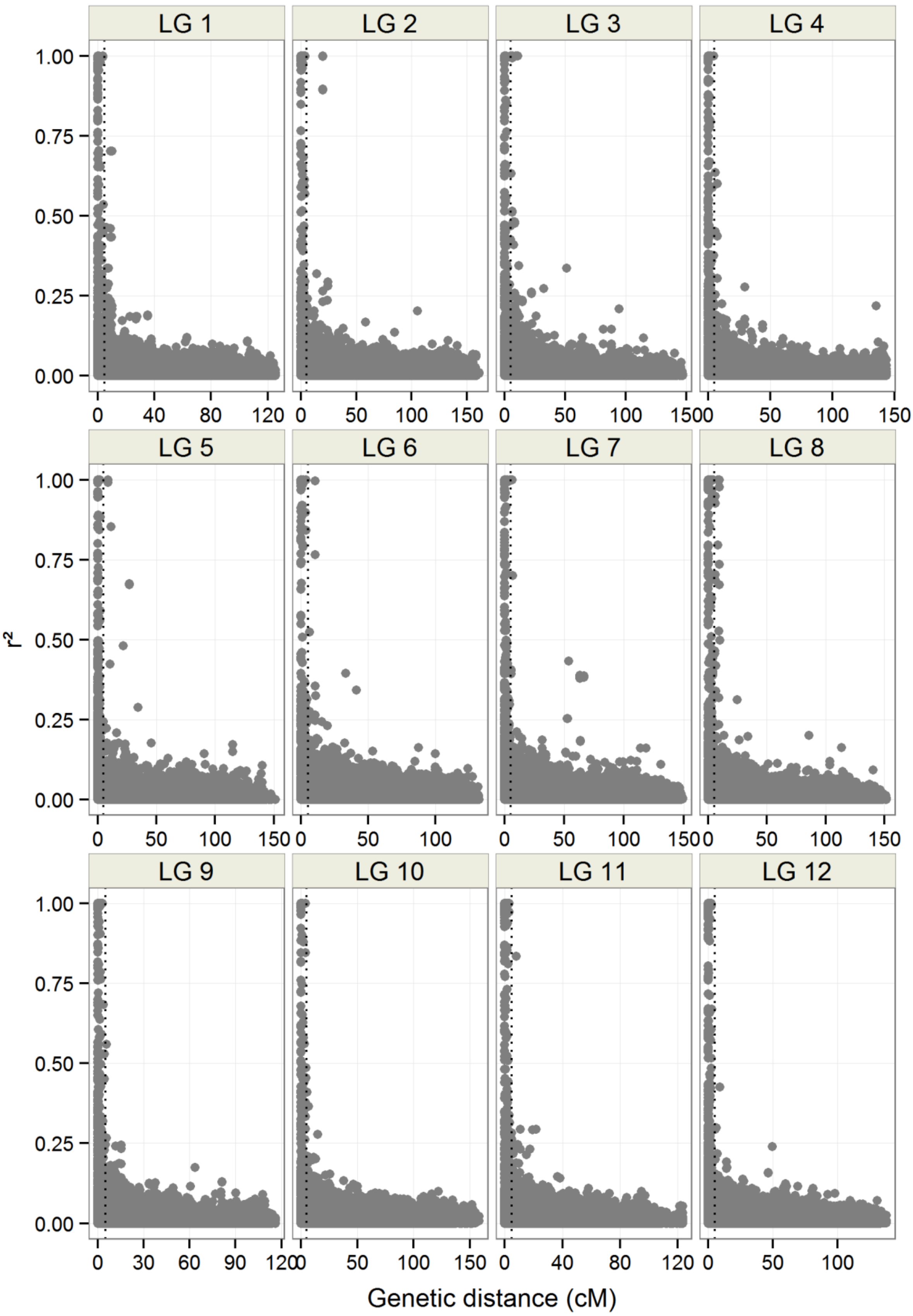

Figure S4 Pairwise linkage disequilibrium (LD) based on 3,962 single-nucleotide polymorphisms mapped onto the twelve linkage groups (LG) of *P. pinaster*. Only loci with minor allele frequencies greater than 0.01 were included in the analysis.

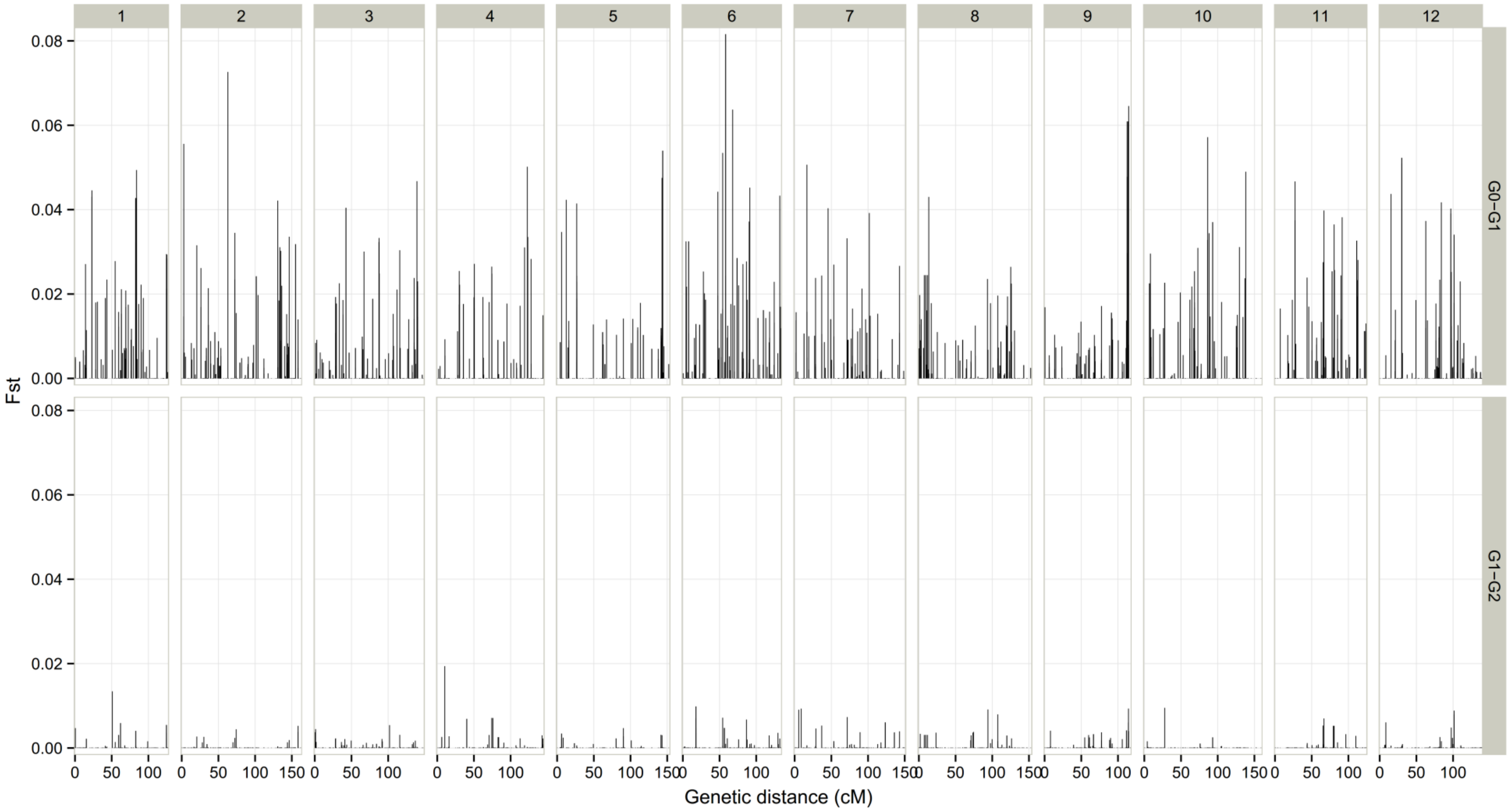

Figure S5 Distribution of the fixation index (F_{ST}) over the 12 chromosome of maritime pine. The top panel represents the F_{ST} between G0 and G1 and the bottom panel represents the F_{ST} between G1 and G2.

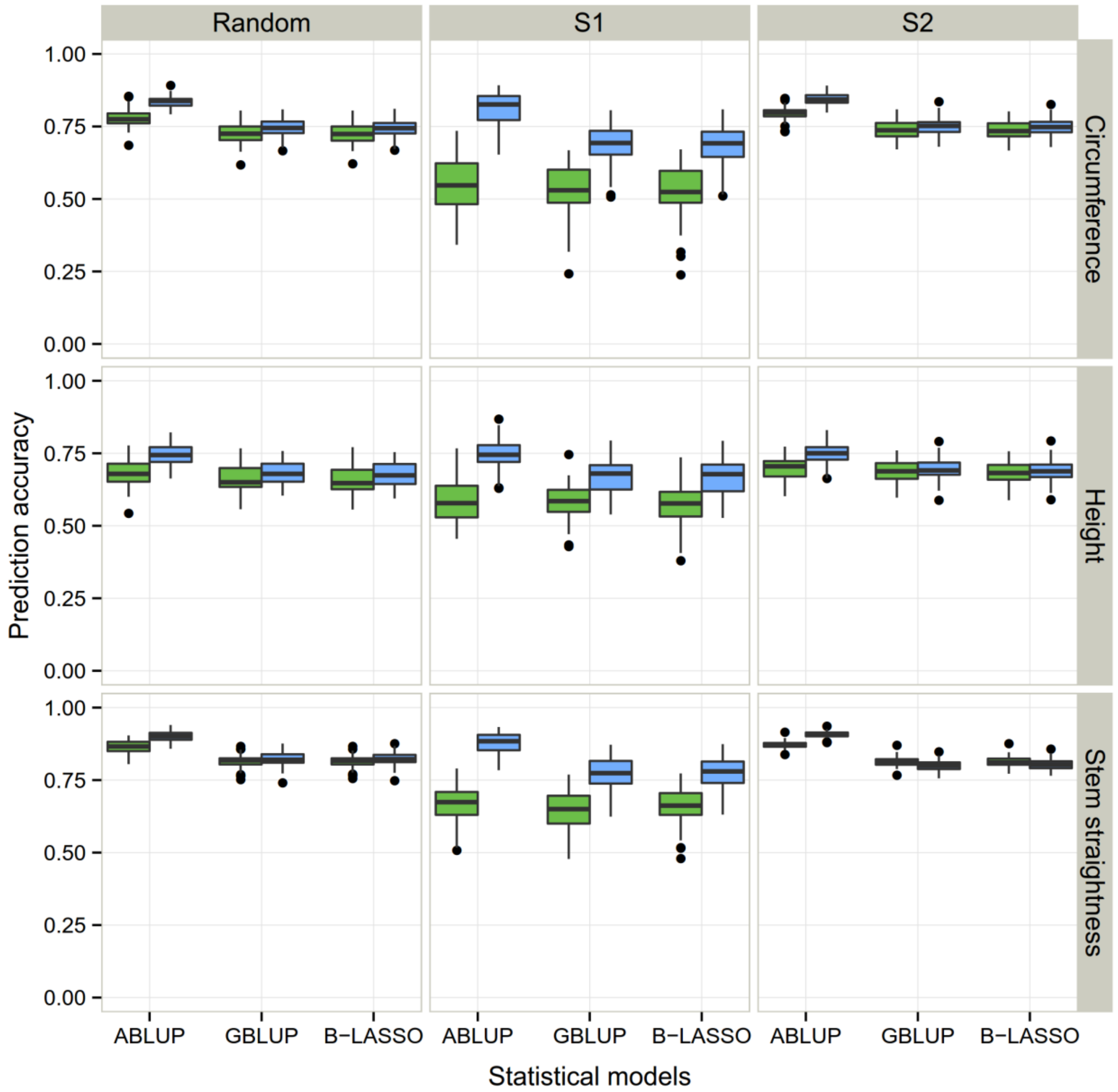

Figure S6 Comparison of prediction accuracies across three sampling and two calibration strategies. Three sampling strategies for the selection of 20% of the G2 population for use as the validation set were used: random, S1: between half-sib families and S2: within full-sib families. Two calibration scenarios were used for each sampling strategy. For predictions for the 20% of the G2 population selected, we used the remaining 80% of the G2 (in green) plus their progenitors (G0 and G1, in blue) as the calibration set. The results for models based on pedigree information (ABLUP) and marker information (GBLUP and B-LASSO), and the results for the three traits studied (tree diameter, height and stem straightness) are presented. The data are represented in a Tukey boxplot.

Table S1 Prediction accuracy and status number (N_S) for four methods of selecting G2 individuals. Prediction accuracy was estimated with three relationship matrices (A_P, A_F and G). Mean values (standard deviation in parentheses) are based on 100 replicates per model. The four selection methods were: Random, HS: half-sib family, FS: full-sib family and CD: coefficient of determination.

	**Prediction accuracy**			**N_S**
	**A_PBLUP**	**A_FBLUP**	**GBLUP**	
Random	0.35 (0.05)	0.51 (0.05)	0.54 (0.05)	20.4 (0.33)
HS	0.37 (0.06)	0.53 (0.06)	0.56 (0.05)	19.8 (0.20)
FS	0.35 (0.05)	0.53 (0.05)	0.55 (0.04)	20.7 (0.24)
CD	0.37 (0.06)	0.54 (0.06)	0.56 (0.06)	25.1 (0.18)

Table S2 Prediction accuracy and bias for the use of the progeny population for validation (calibration set = G0 and G1, validation set= G2). The results for the ABLUP, GBLUP and Bayesian LASSO (B-LASSO) models and for the traits tree circumference, height and stem straightness are presented.

Trait	Method	Prediction accuracy	Bias
Circumference	ABLUP	0.81	1.15
	GBLUP	0.79	1.07
	B-LASSO	0.80	0.99
Height	ABLUP	0.72	1.22
	GBLUP	0.70	1.10
	B-LASSO	0.71	1.07
Stem straightness	ABLUP	0.85	1.36
	GBLUP	0.82	1.21
	B-LASSO	0.83	1.06
